# Complete mitochondrial genome of the Sakhalin sculpin *Cottus amblystomopsis* (Cottoidei: Cottidae)

**DOI:** 10.1080/23802359.2017.1318681

**Published:** 2017-04-24

**Authors:** Evgeniy S. Balakirev, Pavel A. Saveliev, Francisco J. Ayala

**Affiliations:** a Department of Ecology and Evolutionary Biology, University of California, Irvine, CA, USA;; b A.V. Zhirmunsky Institute of Marine Biology, National Scientific Center of Marine Biology, Far Eastern Branch, Russian Academy of Sciences, Vladivostok, Russia;; c School of Natural Sciences, Far Eastern Federal University, Vladivostok, Russia

**Keywords:** Sakhalin sculpin, *Cottus amblystomopsis*, amphidromous life history, Cottidae

## Abstract

The complete mitochondrial genome was sequenced in two individuals of the Sakhalin sculpin *Cottus amblystomopsis*. The genome sequences are 16,526 and 16,527 bp in size, and the gene arrangement, composition, and size are very similar to the other sculpin mitochondrial genomes published previously. The difference between the two genomes studied is low, 0.28%, in spite of the relatively long distance separating the localities. The data are consistent with the amphidromous life history of *C. amblystomopsis*, promoting gene flow even between distantly located rivers.

The Sakhalin sculpin *Cottus amblystomopsis* Schmidt is an amphidromous fish distributed widely in the rivers of the Sea of Japan and the Sea of Okhotsk including Hokkaido Island, Southern Sakhalin Island, and Southern Kuril Islands (Shedko 2001; Nakabo 2002; Chereshnev 2003). Adult sculpins inhabit and spawn in freshwater and their larvae drift downstream to marine environments where they spend about one month, after which they return back to rivers as juveniles (Goto 1990). Kinziger et al. (2005) treated the Sakhalin sculpin *C. amblystomopsis* (along with *C. nozawae,* endemic to the Japan Islands) as a *Cephalocottus* clade or subgenus within the family *Cottidae*. Goto et al. (2015) considered *C. amblystomopsis* within the genus *Cottus* as the lineage C from East Asia. The evolutionary history and phylogenetic relationships of *C. amblystomopsis* remained unclear due to limited genetic data available for the species.

We have sequenced two complete mitochondrial (mt) genomes of *C. amblystomopsis* (GenBank accession numbers KY563345 and KY563346) from the Taranai River (46°37.76′ N; 142°24.31′ E), Sakhalin Island, and the Djigitovka River (44°50.06′ N; 136°06.95′ E), Primorsky krai, Russia, using primers designed with the program mitoPrimer_V1 (Yang et al. 2011). The fish specimens are stored at the museum of the A. V. Zhirmunsky Institute of Marine Biology, National Scientific Center of Marine Biology, Vladivostok, Russia (www.museumimb.ru) under accession numbers MIMB 33262 and MIMB 33263. The genome sequences are 16,526 and 16,527 bp in size and the gene arrangement, composition, and size are very similar to the sculpin fish genomes published previously. We detected 47 single nucleotide and one length differences between the haplotypes CAM1-13 and CAM1-14; total sequence divergence (*D*_xy_) is 0.0028 ± 0.0005.

Comparison of the two mt genomes now obtained with other complete mt genomes available in GenBank for the genera *Cottus* and *Mesocottus* reveals a close affinity of *C. amblystomopsis* to the cluster *C. reinii* +* C. hangiongensis*+* C. koreanus* within the genus *Cottus* (Figure 1). The difference between the two *C. amblystomopsis* mt genomes now studied is low, 0.28%, in spite of relatively long distance separating the localities (∼1670 km along the coast line). The data are consistent with the amphidromous life history of *C. amblystomopsis* (Goto 1990), promoting gene flow between distantly located rivers (Goto & Andoh 1990). The juveniles returning to freshwater environment are suggested to derive from larval pools originating from multiple source rivers, where larval mixing and long-range dispersal result in high connectivity across the coasts of Sakhalin Island and Primorye Territory. Thus, the amphidromous life history of *C. amblystomopsis* might constraint intraspecific divergence as was also shown for other amphidromous fishes (Chubb et al. 1998).

**Figure 1.**
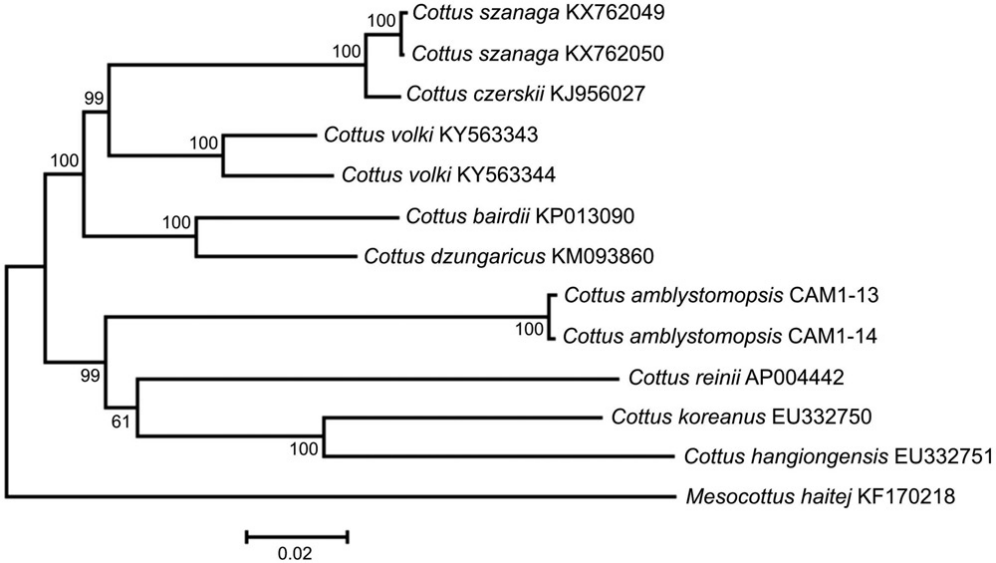
Maximum likelihood tree for the Sakhalin sculpin *Cottus amblystomopsis* specimens CAM1-13 and CAM1-14, and GenBank representatives of the family Cottidae. The tree is constructed using whole mt genome sequences. The tree is based on the General Time Reversible + gamma + invariant sites (GTR + G + I) model of nucleotide substitution. The numbers at the nodes are bootstrap percent probability values based on 1000 replications.
